# Proceedings of the Mississippi Valley Dental Association

**Published:** 1875-07

**Authors:** 


### PROCEEDINGS OF THE MISSISSIPPI VALLEY DENTAL ASSOCIATION.

The next subject considered was senstive dentine.

Dr. Taft: This condition is usally found at a point of decay. Sometimes however decaying dentine exhibits no increase of sensitiveness at all and yet retains its vitality, in other cases no sensitiveness is apparent because the decaying tissue becomes devitalized in advance of the decay. Sensitiveness exists sometimes where their is no decay. It occurs in a marked degree at the necks of some teeth. In the cavity of decay it is sometimes confined to a small point, or to the periphery of the dentine, or to a thin stratum of dentine immediately underneath that which is decaying. It varies in intensity and in susceptibility to the influencc of exciting agents. Where the vital force is feeble, sensitiveness is frequently not manifested because death of the structure occurs in advance of decay. In some cases there is but little or apparently no exalted sensitiveness, though devitalization does not preceed the decomposition. This condition exists either because of the vigorous vitality and consequent resistance to irritation, or it sometimes is dependent upon the character of the agent producing the decay. Sensitiveness of the dentine does not occur as persistent and prolonged pain as in soft tissues, unless the irritating agent be continously applied.

When sensitiveness of the dentine is confined to a thin layer in a cavity of decay, it may readily be removed by excavation, but if it extends deeply this is not practicable.

Arsenious acid should never be used to destroy sensitiveness of the dentine, even if it should not ultinately destroy the pulp.

Local treatment of any kind is not always effective especially in actively inflammatory conditions. Constitutional treatment will often be required. Simply to remove all irritants and close up the cavity with some non-conducting substance, leaving nature to remedy the condition, is sometimes all that is required. Escharotics, carbolic acid, nitrate of silver and chloride zinc, are ofter used. Chloride of zinc does not act uniformly, its effect is sometimes of temporary duration. I have used the "Dental Pain Obtunder," a new remedy, for five or six months. In many cases it operates well.

Dr. Morgan: The application of iodine or any mild escharotic will often relieve sensitiveness about the necks of teeth. Filling the interstices between them with prepared chalk and allowing it to remain over night often accomplishes the object. I have heard that sulphuric acid is a good remedy, I am unwilling to employ so powerful an agent.

Several gentlemen then related their experience with the "Dental Pain Obtunder," some reported favorably and others unfavorably of it.

The subjects, "Dental Hygeine" and "Treatment of exposed pulps" were considered in conjunction.

Dr. Cravens, who introduced the practice of capping pulps with lacto-phosphate of lime, was called upon to explain his process and theory.

Dr. Cravens: The powdered phosphate of lime can be obtained at any drug-store, it is nearly insoluble. Lactic acid is a solvent of phosphate lime but it has little effect on the powdered phosphate. The combination of the two is mechanical not chemical. My idea is to digest the phosphate lime in the lactic acid and by placing the compound in contact with the pulps I expect to effect an assimilation between the plastic lymph or liquor sanguinis, thus forming a clot. My practice is to cut the pulp slightly causing a drop of blood to flow out, this forms a clot. Place a bit of bibulous paper aganist this and you find it is stained with a yellowish fluid which you recognize as the plastic lymph. It is expected that the lacto-phosphate of lime will unite with this lymph to form a covering over the exposed pulps. I have been asked whether carbonate of lime is not formed? I do not know. It is immaterial to me.

As a result of the application I find a new deposit which covers the pulps just to the extent of the exposure. Examination proves that this is not simply hardened phosphate of lime but a new substance. It is a transparent or semi-translucent substance resembling glue. It is entirely different from this overlaying substance which I have remove. The general continuity of surface in the cavity is preserved. The line of union of the dentine and the formation is apparent to the eye, but can not be distinguished by the touch of an instrument.

The phosphate of lime deteriorates when exposed, the lactic acid precipitates in flocculi. A superior preparation of the lacto-phosphate has been imported from Berlin.

The lactic acid applied in a sensitive cavity and protected with a covering of oxy-chloride of zinc soon effects a cure of the sensitiveness. The dentine will be found to present a peculiar flinty appearance.

Dr. Cravens, in conclusion, gave a detailed account of three cases in which he had capped exposed pulps, *none* of which had resulted unfavorably.

Dr. Reed spoke of two instances in which he had used the lacto-phosphate without success. Nevertheless he thinks it may prove valuable. Dr. Taft treated unsuccessfully a tooth for him (Reed). Dr. Jay thought the experiment thus far had proved rather unsatisfactory.

Dr. Rehwinkle spoke hopefully of the new remedy and said he did not feel ready to condemn any new theory or method of practice which was not manifestly impracticable, until he has made thorough investigation and trial of it. We often make mistakes in the selection of a specific, The fault is often attributable to our errors of judgment rather than to the inefficiency of the remedy.

The preserving of an exposed pulp, when there is not a state of inflammation or diseased condition is ordinarily a simple matter requiring only delicacy of manipulation to insure success. Dr. Watt's, method of capping with a solution of gutta percha and chloroform is excellent. Do not think it possible to preserve the pulps alive when suppuration has commenced.

He hailed the announcement of Dr. Craven's discovery with delight when that announcement was first made in the Journal, believing his method of treatment be a rational one.

He regards the phosphate of lime as a valuable agent to remedy the softened chalky condition of teeth that is so frequently met.

Dr. Watt: The result of inflammatory action in the exposed pulp is, a throwing out of a plasm which is organic matter. Lactic acid will do several things. It will arrest decomposition and operate as an antiseptic. It will dissolve away the debris. The phosphate of lime I regard merely as a vehicle to carry the acid to the parts. In the preparation of lacto-phosphate of lime we have a mechanical mixture, sub-phosphate of lime with a solution of phosphate of lime in lactic acid. Provided the two would remain together, is that solution of any use? Would it accomplish the same result? I think not quite. Supposing the lactic acid lets go the sub-phosphate with which it is already combined. As particle after particle is released it acts on the deteriorated foreign matter of the pulp, in its nascent condition in producing its action more pomptly and with less pain.

It is I think, the lactic acid which produces the result.

Dr. Jay inquired what course should be pursued where the pulp is strangulated and protrudes through a narrow orifice into the cavity, presenting a prominence.

Dr. Watt: In such cases I have applied the rubber dam and thrown a fine jet of rhigolene upon the projecting part, almost freezing it, on the principle of reducing strangulated hernia. Sometimes I have dropped snow or a small particle of ice into the cavity then appled a little carbolic acid. The combination produces a most intense cold, the carbolic acid, because of its affinity for water, melting the ice rapidly. The difficulty is that as the part has been only recently strangulated there is danger of too much destruction of tissue.

Dr. Craverns asked what property it is of this preparation which allay sensitiveness of the dentine. Dr. Watt replied that the result was mainly due to the lactic acid. Said he was a believer in the theory of nerve fibrils permeating the dentine. They are dissolved in the acid. The gelatinous portion of the dentine becomes hard like horn under the action of the acid.

Dr. Taft said he had been experimenting with this preparation for about a year with encouraging results. Had not always met with success. Some persons never indorse a theory or feel encouraged in their investigations unless their experiments are uniformly and invariably successful. In the case of Dr. Reid there was a complication. The periosteum as well as the pulp was diseased. He has always thought that if circumstances had favored the devoting of proper attention to the treatment of the case, the pulp could have been saved.

He related a case of a gentleman for whom he had treated an exposed aching pulp six or seven months ago. He cleaned out the cavity as well as possible, and applied the lacto phosphate of lime, sealing the cavity with wax. Although it had been aching severely for three days, it now ceased at once. The patient returned in three days and said he had not suffered any pain. He would not have known that it had ever ached. Made a second application with the same result.

Then applied carbolic acid as an experiment and sealed up with wax. Afterward applied the lacto-phosphate for the third time.

Noticed that the pulp which at first was of a deep red color, had changed to a rose color. After the fourth application he fillled with oxy-chloride of zinc and that filling remains to the preseut time. The tooth has never given any uneasiness. This is only one of several similar cases. Some would say the pulp is dead in this case. There is no indication of it.

He has lost two cases out of twenty or more.

